# *Circ*ERCC2 ameliorated intervertebral disc degeneration by regulating mitophagy and apoptosis through miR-182-5p/SIRT1 axis

**DOI:** 10.1038/s41419-019-1978-2

**Published:** 2019-10-03

**Authors:** Lin Xie, Weibo Huang, Zhenhua Fang, Fan Ding, Fei Zou, Xiaosheng Ma, Jie Tao, Jingkang Guo, Xinlei Xia, Hongli Wang, Zuochong Yu, Feizhou Lu, Jianyuan Jiang

**Affiliations:** 1 Department of Orthopedics, Huashan Hospital, Fudan University, 12 Mid-Wulumuqi Road, Shanghai, 200040 China; 2 Department of Orthopedic Surgery, Wuhan Fourth Hospital, Huazhong University of Science and Technology, 473 Hanzheng Street, Wuhan, 430000 China; 3 Department of Spinal Surgery, Wuhan Puren Hospital, Wuhan University of Science and Technology, 1 Benxi Street, Wuhan, 430080 China; 4 Laboratory of Neuropharmacology and Neurotoxicology, Shanghai University, 381 Nanchen Road, Shanghai, 200436 China; 5 Department of Orthopedic Surgery, Jinshan Hospital, Fudan University, 1508 Longhang Road, Shanghai, 201508 China; 6 Department of Orthopedic Surgery, The Fifth People’s Hospital of Shanghai, Fudan University, 128 Ruili Road, Shanghai, 201100 China

**Keywords:** Mitophagy, miRNAs, RNAi

## Abstract

The molecular mechanism of intervertebral disc degeneration (IVDD) remains unclear. This study aimed to investigate the role of circular RNAs (circRNAs) in the pathogenesis of IVDD. We sued nucleus pulposus (NP) tissues of patients, tert-butyl hydroperoxide (TBHP) stimulated NP cells (NPCs), and IVDD rat model to explore the interaction between *circ*ERCC2 and miR-182-5p/SIRT1 axis. The results showed that downregulation of *circ*ERCC2 increased the level of miR-182-5p and decreased the level of SIRT1 in degenerative NP tissues in vivo as well as in TBHP-stimulated NPCs in vitro. Treatment of SIRT1-si activated apoptosis and inhibited mitophagy. Moreover, miR-182-5p-si could regulate the mitophagy and the apoptosis of NPCs by targeting SIRT1. The effects of *circ*ERCC2 on NPCs and IVDD rat model were mediated by miR-182-5p/SIRT1 axis. In conclusion, this study provides the first evidence that *circ*ERCC2 could ameliorate IVDD through miR-182-5p/SIRT1 axis by activating mitophagy and inhibiting apoptosis, and suggests that *circ*ERCC2 is a potentially effective therapeutic target for IVDD.

## Introduction

Low back pain (LBP) causes high medical costs and socioeconomic burden. It has been reported that up to 80% of the population suffers from LBP and 10% of them become chronically disabled^1^. Although the pathogenesis of LBP is poorly understood, intervertebral disc degeneration (IVDD) has been proposed to be the major cause of LBP^2,3^. IVDD is characterized by increased oxidative stress, the degradation of extracellular matrix (ECM) and apoptosis, and decreased autophagy or mitophagy^4,5^. Given poor understanding of the pathogenesis of IVDD, current strategies for IVDD treatment are not satisfying.

The intervertebral disc is composed of three parts, i.e. upper endplate, center nucleus pulposus (NP) and outer annulus fibrosus (AF)^6,7^. The main cells in the NP tissues are NP cells (NPCs), which play important roles in ECM degradation^7,8^. In IVDD, NPCs are dysfunctional during the progression of IVDD, causing excessive production of proinflammatory molecules^9–14^. The abnormal activities of NPCs could accelerate IVDD. Therefore, it is important to inhibit the abnormal activities of NPCs to ameliorate IVDD^4,15,16^.

Circular RNA (circRNA) is a large endogenous class of non-coding RNA which forms a closed loop structure with 5′ and 3′ ends joining together. Some circRNAs act as sponges for miRNAs and possess many binding sites for miRNAs to regulate the expression of the target mRNAs as RNA-induced silence complex (RISC). Accordingly, cell metabolism, differentiation, proliferation, and survival involving these targeted mRNAs will be affected due to the biding of circRNAs and miRNAs^17^. Increasing evidence suggests the role of circRNAs in the pathogenesis of IVDD^18,19^. This study aimed to investigate the role of circRNAs in the pathogenesis of IVDD, and we selected *circ*ERCC2 based on bioinformatics analysis and explored its role in the regulation of mitophagy and apoptosis during the progression of IVDD.

## Results

### *Circ*ERCC2 was downregulated in IVDD and regulated mitophagy and apoptosis

**Fig. 1:**
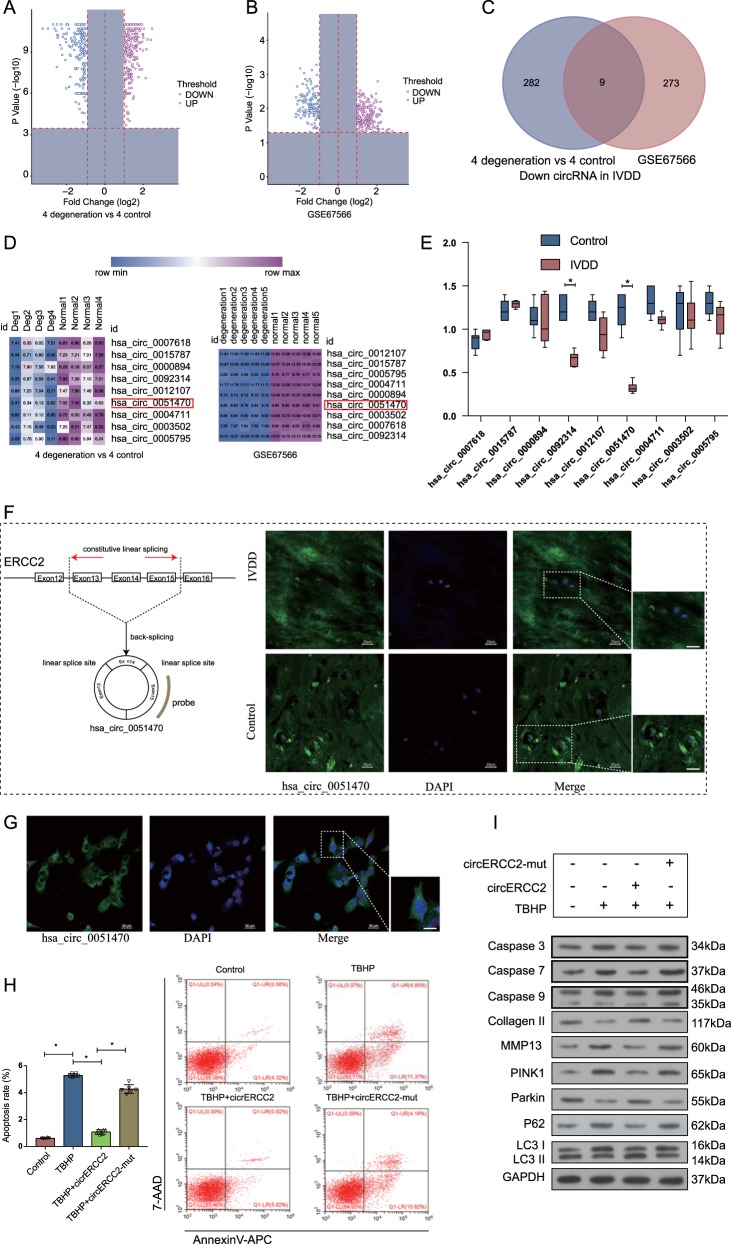
*Circ*ERCC2 was downregulated in IVDD and regulated mitophagy and apoptosis. **a** Volcano plots showed differential expression of circRNAs detected by circRNA microarray in IVDD compared with the control. **b** Volcano plots showed differential expression of circRNAs in GSE67566. **c** The 9 downregulated circRNAs in IVDD were identified based on the overlap of circRNA microarray and GSE67566. **d** Heatmap of 9 circRNAs in circRNA microarray and heatmap of 9 circRNAs in GSE67566. **e** qRT-PCR analysis confirmed the downregulation of circRNAs in IVDD compared with control. *p < 0.05. **f** *circ*ERCC2 is transcribed from 13, 14, and 15 exons of the ERCC2 gene. The expression of *circ*ERCC2 was lower in NP tissues from IVDD compared with the control detected by FISH. **g** FISH detection of *circ*ERCC2 in the cytoplasm of NPCs. In (**f**) and (**g**), blue fluorescence indicated the nucleus and green fluorescence indicated *circ*ERCC2. Scale bar: 20 μm. **h** Representative plots of apoptosis detected by flow cytometry. *circ*ERCC2 inhibited the rate of apoptosis of NPCs. *p < 0.05, **p < 0.01. **i** NPCs were treated by TBHP or/and *circ*ERCC2, and mitophagy and apoptosis related proteins were detected by Western blot analysis

Identification of differentially expressed circRNAs was performed by overlapping microarray analysis of human circRNAs (Arraystar, CA, USA) and microarray dataset (GSE67566) obtained from Gene Expression Omnibus (GEO) database. Nine circRNAs downregulated in IVDD were analyzed (Fig. 1a–d). Quantitative real-time PCR (qRT-PCR) was used to confirm the downregulated circRNAs in the degenerative NP tissues from patients with IVDD and nondegenerative NP tissues from patients with Hirayama disease. We found that hsa_circ_0051470 (*circ*ERCC2) was downregulated in IVDD (Fig. 1e). Furthermore, *circ*ERCC2 was downregulated in IVDD based on RNA fluorescence in situ hybridization (FISH) (Fig. 1f). The expression of *circ*ERCC2 was also detected in rat NPCs (Fig. 1g). The transfection of *circ*ERCC2 inhibited the rate of apoptosis of NPCs (Fig. 1h). In addition, Western blot analysis showed that *circ*ERCC2 inhibited apoptosis and regulated mitophagy induced by tert-butyl hydroperoxide (TBHP) treatment in NPCs (Fig. 1i).

### miR-182-5p was upregulated in IVDD and regulated NPCs mitophagy and apoptosis

**Fig. 2:**
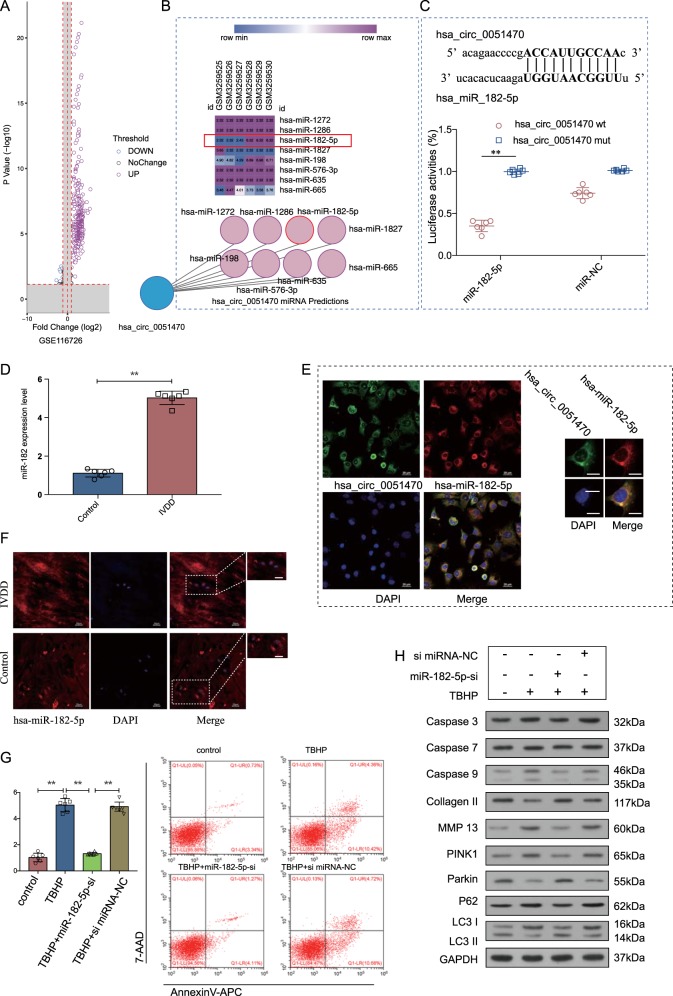
miR-182-5p was upregulated in IVDD and regulated mitophagy and apoptosis. **a** Volcano plot showed differential expression of miRNAs in GSE116726. **b** The predicted 8 miRNAs of *circ*ERCC2 (has_circ_0051470) and heatmap of the 8 miRNAs in GSE116726. (**c**) NPCs were transfected with miR-182-5p and luciferase constructs of *circ*ERCC2 containing wild-type putative miR-182-5p binding sites or mutated sites. *p < 0.05, **p < 0.01. **d** qRT-PCR analysis confirmed the upregulation of miR-182-5p in the degenerative NP samples from patients with IVDD compared with the control. *p < 0.05, **p < 0.01. **e** FISH showed that both *circ*ERCC2 and miR-182-5p were located in the cytoplasm. Blue fluorescence indicated the nucleus, green fluorescence indicated *circ*ERCC2, and red fluorescence indicated miR-182-5p. Scale bar: 20 μm. **f** FISH analysis of miR-182-5p in NP samples from patients with or without IVDD. Blue fluorescence indicated the nucleus, and red fluorescence indicated miR-182-5p. Scale bar: 20 μm. **g** Representative plots of apoptosis detected by flow cytometry. miR-182-5p-si inhibited apoptosis induced by TBHP in NPCs. *p < 0.05, **p < 0.01. **h** NPCs were treated by TBHP or/and miR-182-5p-si, and mitophagy and apoptosis related proteins were detected by Western blot analysis

A microarray dataset (GSE116762) was used to establish the differential expression of miRNAs. The expression of 531 miRNAs was increased in IVDD compared with the controls (the criteria of mean fold change > 2.0 and *p* values < 0.05) (Fig. 2a). Targets of *circ*ERCC2 were predicted by circRNA online tool (http://circinteractome.nia.nih.gov/bin/circsearchTest)^20^. The 531 miRNAs were compiled with the predicted target miRNAs and miR-182-5p was selected as the candidate miRNA (Fig. 2b). The binding sites of miR-182-5p to *circ*ERCC2 were validated via the dual-luciferase assay (Fig. 2c). qRT-PCR conformed the expression of miR-182-5p in NP tissues from patients with IVDD or hirayama disease (non-degenerative) (Fig. 2d). Subcellular localization of circRNAs and miRNAs was used to determine their mode of action. FISH showed that *circ*ERCC2 and miR-182-5p were both located in the cytoplasm (Fig. 2e). Moreover, the expression of miR-182-5p was upregulated in IVDD compared to the control (Fig. 2f). The treatment of miR-182-5p-si inhibited apoptosis induced by TBHP in NPCs (Fig. 2g). Moreover, miR-182-5p-si attenuated the effect of TBHP on apoptosis and mitophagy of NPCs (Fig. 2h).

### Identification of SIRT1 as a target of miR-182-5p and miR-182-5p/SIRT1 axis as a target of *circ*ERCC2

**Fig. 3:**
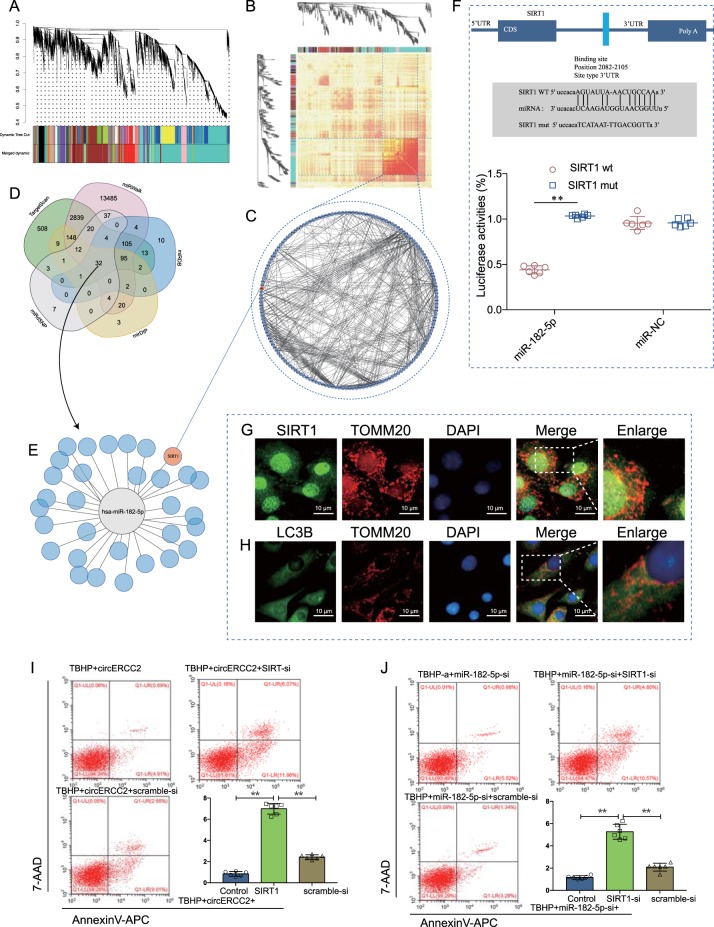
Identification of SIRT1 as a target of miR-182-5p. **a** Weighted correlation network analysis (WGCNA) of the GEO database (GSE27494, GSE34095, GSE41883, and GSE15227). The topological overlaps of mRNA and their relations to modules were shown in dendrogram. **b** The turquoise module. **c** A graphic depiction of the turquoise module using String (https://string-db.org/). **d** Venn diagram showing targets by different algorithms. **e** Cystoscope was employed to confirm the targets of miR-182-5p. **f** NPCs were transfected with miR-182-5p and luciferase constructs of SIRT1 containing wild-type putative miR-182-5p binding sites or mutated sites. *p < 0.05, **p < 0.01. Immunofluorescence double staining for co-localization of SIRT1 (**g**) and LC3B (**h**) with TOMM20 in NPCs. **i** Representative plots of apoptosis detected by flow cytometry. SIRT1-si decreased apoptosis inhibition of *circ*ERCC2 in NPCs. *p < 0.05, **p < 0.01. **j** SIRT1-si decreased a*p*optosis inhibition of miR-182-5p-si in NPCs. *p < 0.05, **p < 0.01

**Fig. 4:**
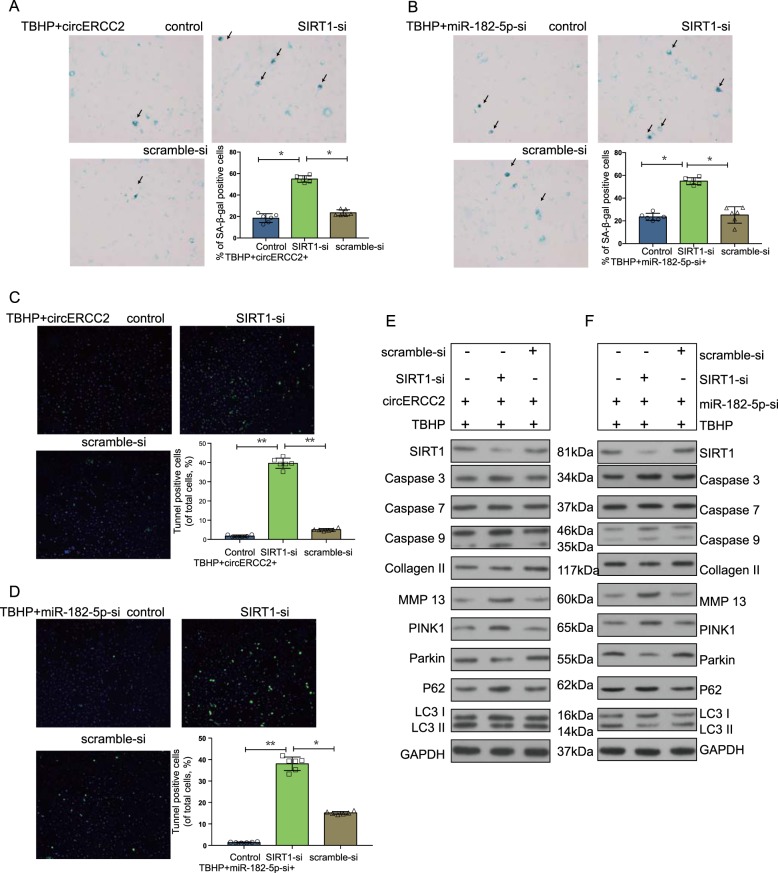
miR-182-5p/SIRT1 axial is the target of *circ*ERCC2. **a** The cell senescence was determined using SA-β-gal staining. SIRT1-si decreased senescence inhibition of *circ*ERCC2 in NPCs. *p < 0.05, **p < 0.01. **b** SIRT1-si decreased senescence inhibition of miR-182-5p-si in NPCs. *p < 0.05, **p < 0.01. **c** Cell apoptosis was determined by TUNNEL staining. SIRT1-si abolished the inhibitory effect of *circ*ERCC2 on the apoptosis of NPCs. *p < 0.05, **p < 0.01. **d** SIRT1-si abolished the inhibitory effect of miR-182-5p-si on the apoptosis of NPCs. *p < 0.05, **p < 0.01. **e** SIRT1-si antagonized protective effects of *circ*ERCC2 on NPCs. **f** SIRT1-si antagonized protective effect of miR-182-5p-si on NPCs

A weighted gene co-expression network analysis (WGCNA) analysis was performed on microarray datasets (GSE27494, GSE34095, GSE41883 and GSE15227) from the GEO database. The topological overlaps of mRNA and the relation to modules were shown in dendrogram. A graphic depiction of the turquoise module using String (https://string-db.org/) was shown (Fig. 3a, c). The Venn diagram predicted that miR-182-5p targeted SIRT1 with different algorithms. Cystoscope was employed to determine the target of miR-182-5p (Fig. 3d, e). The binding sites were evaluated by dual-luciferase activity (Fig. 3f). Double staining of SIRT1, LC3B and TOMM20 (mitochondrial membrane protein marker) showed the action mode in NPCs (Fig. 3g, h). SIRT1-si decreased apoptosis of NPCs which were inhibited by *circ*ERCC2 (Fig. 3i) and miR-182-5p-si (Fig. 3j). Moreover, SIRT1-si blocked inhibitory effect of *circ*ERCC2 on the senescence of NPCs (Fig. 4a), and decreased inhibitory effect of miR-182-5p-si on the senescence of NPCs (Fig. 4b). SIRT1-si also decreased inhibitory effects on the apoptosis of NPCs by *circ*ERCC2 (Fig. 4c) and miR-182-5p-si (Fig. 4d). Western blot analysis of SIRT1, NPCs apoptosis (caspase3, caspase7 and caspase9), ECM degradation (MMP13 and collagen II) and NPCs mitophagy (PINK1, PARKIN, P62, and LC3II/I) showed that SIRT1-si antagonized protective effects of both *circ*ERCC2 (Fig. 4e) and miR-182-5p-si (Fig. 4f) on NPCs.

### *circ*ERCC2 alleviated IVDD in a rat model

**Fig. 5:**
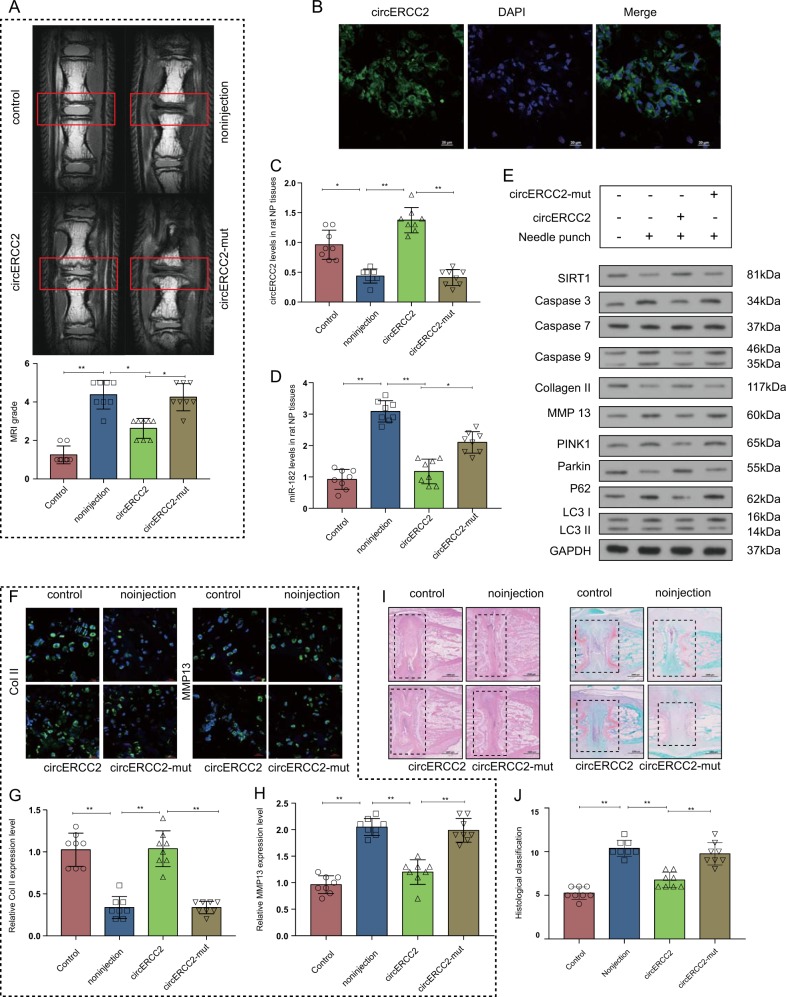
*Circ*ERCC2 ameliorated IVDD in vivo. **a** T2-weighted MRI of rat tail with punctured disc. MRI grade was significantly lower in *circ*ERCC2 group. *p < 0.05, **p < 0.01. **b** FISH showed that *circ*ERCC2 expression was located in the NP region. Blue fluorescence indicated the nucleus and green fluorescence indicated *circ*ERCC2. Scale bar: 20 μm. **c** *circ*ERCC2 in IVDD was upregulated in the *circ*ERCC2 group. *p < 0.05, **p < 0.01. **d** miR-182-5p level in IVDD decreased following the injection of *circ*ERCC2. *p < 0.05, **p < 0.01. **e** *circ*ERCC2 inhibited ECM degradation, induced mitophagy and inhibited apoptosis in vivo. **f**–**h** Immunofluorescence staining showed upregulated collagen II and downregulated MMP13 in *circ*ERCC2 group. Scale bar: 25 μm. **i** H&E staining and Safranin-O/fast green staining showed that IVDD was ameliorated in *circ*ERCC2 group. Scale bar: 1000 μm. **j** The histological grades were significant decreased at week 8 in *circ*ERCC2 group. *p < 0.05, **p < 0.01, *n* = 6

We reviewed the T2-weighted MRI results of rat tails with punctured disc. The MRI grade was significantly lower in *circ*ERCC2 group compared with non-injection group at 8 weeks (Fig. 5a). FISH showed that *circ*ERCC2 was located in the NP region of rat disc tissues (Fig. 5b), and qRT-PCR showed that the increased levels of miR-182-5p in IVDD were changed by the injection with *circ*ERCC2 (Fig. 5c, d). Moreover, *circ*ERCC2 injection alleviated IVDD through enhancing mitophagy response, and reducing apoptosis and ECM degradation in the rat model of IVDD (Fig. 5e). Immunofluorescence showed that *circ*ERCC2 injection changed the expression of collagen II and MMP13 in the rat model of IVDD (Fig. 5f–h). In control group, most of the space in the discs was occupied by NP tissues whose volume was considerably large. NPCs were uniformly dispersed among the matrix. The rest space was well-organized AF. Compared to e control group, the volume of NP tissue in IVDD group was smaller. NPCs were aggregated and divided by proteoglycan matrix, indicating serious degeneration of NPCs. However, compared to IVDD group, *circ*ERCC2 treatment effectively alleviated the degeneration of NPCs as well as the disorganization and fibrosis of AF. Furthermore, Safranin-O staining showed decreased volume of proteoglycan matrix in IVDD group, abundant proteoglycan matrix in control group, and NP tissues of *circ*ERCC2 treatment group showed less proteoglycan decrease compared to IVDD group (Fig. 5i). In addition, histological grades of *circ*ERCC2 group were lower than IVDD group at week 8 (Fig. 5j). Collectively, these results suggested that *circ*ERCC2 alleviated IVDD.

## Discussion

The precise molecular mechanism underlying IVDD remains elusive. CircRNAs play fundamental roles in a variety of physiological functions^17,21,22^. Unlike the traditional linear RNAs, circRNAs have a closed circular structure and are not affected by RNA exonuclease so that their expression is more stable and less prone to degradation^23^. Several studies have confirmed that circRNAs are rich in miRNA binding sites and act as miRNA sponges to abolish the inhibition of miRNAs on their target genes in a mechanism called the competitive endogenous RNA (ceRNA)^24,25^. Increasing evidence indicates that circRNAs act as miRNA inhibitors in the development and progression of IVDD^18,19^.

In present study, *circ*ERCC2 was first identified to be downregulated in IVDD. Bioinformatics analysis revealed that *circ*ERCC2 contains miR-182-5p target sites, which was verified by dual-luciferase analysis. In addition, the effects of *circ*ERCC2 can be inhibited by SIRT1-si. Therefore, we proposed that the effects of *circ*ERCC2 are mediated by miR-182-5p/SIRT1 axis. The present study showed that the overexpression of *circ*ERCC2 significantly decreased apoptosis, increased mitophagy and decreased ECM degradation of NPCs under TBHP treatment, suggesting that *circ*ERCC2 is beneficial for NPCs survival under oxidative stress. Accordingly, the overexpression of *circ*ERCC2 alleviated IVDD in the rat model in vivo. These data indicate that the downregulation of *circ*ERCC2 may contribute to IVDD progression, and suggest that *circ*ERCC2 is a promising therapeutic target for IVDD.

Several miRNAs can regulate the development of IVDD^26–28^. Although miR-182-5p has been associated with a variety of human diseases^29,30^, this is the first study to identify miR-182-5p as a key factor in IVDD. We found that miR-182-5p could promote apoptosis and reduce mitophagy, and these effects were related to the regulation of *circ*ERCC2 on miR-182-5p/SIRT1 axis. Our results showed that *circ*ERCC2 could target miR-182-5p/SIRT1 axis to inhibit the development of IVDD.

Our previous studies demonstrated that SIRT1 has a protective role in IVDD^31,32^. Furthermore, several studies revealed that SIRT1 plays a key role in mitophagy and apoptosis in a variety of aging-related diseases via SIRT1-Parkin-Mitohphagy pathway^33–37^. In present study, we showed that *circ*ERCC2 regulated the expression of SIRT1 by sponging miR-182-5p. In addition, the ability of SIRT1-si to decrease the anti-apoptotic and mitophagy function of *circ*ERCC2 or miR-182-5p-si confirmed that SIRT1 is a direct target of *circ*ERCC2 and miR-182-5p-si in NPCs. These findings indicate that *circ*ERCC2 may have protective effects on NPCs by regulating mitophagy and apoptosis.

Oxidative stress-induced mitochondrial dysfunction is implicated in the pathogenesis of IVDD^38,39^. The rupture of AF activates oxidative stress, immune response and apoptosis in NPCs^40^. Therefore, in this study TBHP was used to induce oxidative stress in NPCs, and AF was disrupted to induce IVDD in needle-punctured rat model. Mitophagy is a selective autophagy process that regulates cellular metabolism by specifically degrading damaged or redundant mitochondria in the cells^41–43^. Mitophagy has been associated with the progression of several diseases^44–47^. PINK1/Parkin mitophagy pathway has been identified as a classical pathway involved in mitophagy^48^. Zhang et al. reported that Parkin was involved in the pathogenesis of IVDD and may be potential therapeutic target for IVDD^49^. PINK1, Parkin and LC3 II are key proteins for mitophagy initiation, and p62 is indispensable for autophagic degradation^50^. We used these proteins as the markers to evaluate mitophagy. We found that LC3 II and Parkin expression was increased after *circ*ERCC2 treatment, meanwhile, p62 was decreased after *circ*ERCC2 treatment, suggesting that indicating that mitophagy was activated. Therefore, we proposed that *circ*ERCC2 may alleviate IVDD via promoting mitophagy.

There are several limitations of our study. Firstly, although our results support that *circ*ERCC2 could ameliorate IVDD by regulating mitophagy and apoptosis through miR-182-5p/SIRT1 axis, the particular relationship between mitophagy, apoptosis and ECM degradation in NPCs remains unclear. Secondly, the mechanism for the downregulation of *circ*ERCC2 during IVDD process remains unclear. Further investigations are needed to gain deeper understanding of the role of *circ*ERCC2 in IVDD.

**Fig. 6:**
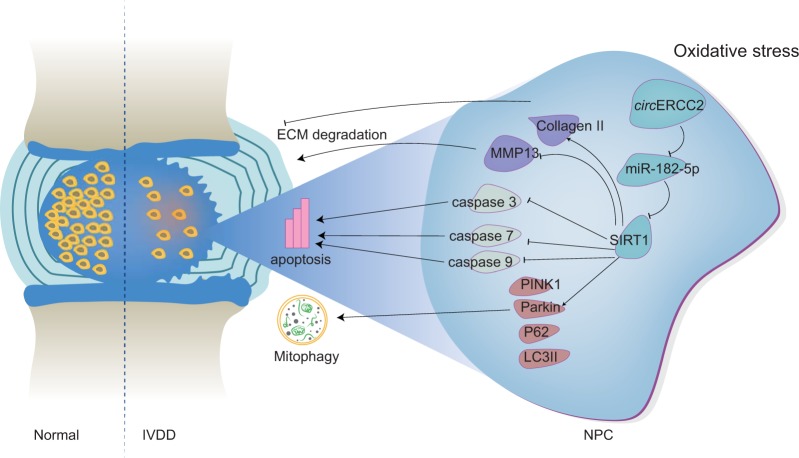
Schematic representation of the mode of action of *circ*ERCC2. *Circ*ERCC2 ameliorates IVDD through targeting miR-182-5p/SIRT1 axis to regulate mitophagy and apoptosis

In summary, this study demonstrates that *circ*ERCC2 can regulate TBHP-induced NPCs apoptosis, mitophagy and ECM degradation via targeting miR-182-5p/SIRT1 (Fig. 6). These findings provide a better understanding of the mechanism involved in the pathogenesis of IVDD and help develop potentially effective therapeutic strategy for IVDD.

## Materials and methods

The entire experimental protocol is schematized in Supplementary Fig. 1.

### Ethics statement

The Ethics Committee of Fudan University Huashan Hospital approved the study protocol, and informed consent was obtained from each donor. The Animal Care and Use Committee of Shanghai University approved the surgical interventions, treatments, and postoperative animal care procedures.

### NP tissues collection

The degenerative NP tissues were obtained from 16 patients undergoing anterior cervical discectomy and fusion (ACDF) due to degenerative cervical disc disease. The control NP tissues were obtained from 16 patients undergoing ACDF due to Hirayama disease (Supplementary Table S1). Of all the samples, 4 control and 4 IVDD samples were used to detect circRNAs using a human circRNA microarray assay. The other samples were used for FISH and qRT-PCR analysis.

### CircRNAs microarray and bioinformatics analysis

CircRNA microarray expression profiles were obtained from human degenerated and non-degenerated intervertebral disc NPCs. The gene expression profile dataset GSE67566 was downloaded from the GEO database. The two-microarray expression were compiled through Venn analysis. The targets of *circ*ERCC2 were then predicted via a circRNA online tool (http://circinteractome.nia.nih.gov/bin/circsearchTest)^20^. Meanwhile, the gene expression profile dataset GSE116726 was downloaded from the GEO database. The predicted targets of *circ*ERCC2 and the upregulated miRNA expression of GSE116726 were compiled. The mRNA targets of miR-182-5p were predicted using five programs: TargetScan (http://www.targetscan.org/vert_71/)^51^, miRWalk (http://mirwalk.umm.uni-heidelberg.de/)^52^, miRDB (http://www.mirdb.org/index.html)^53^, mirDIP (http://ophid.utoronto.ca/mirDIP/)^54^, miRdSNP (http://mirdsnp.ccr.buffalo.edu/index.php)^55^. Then, the gene expression profile datasets GSE27494, GSE41883, GSE15227 and GSE34095 were downloaded from the GEO database. Weighted correlation network analysis (WGCNA) was used to analyze the data of four combined microarrays. 350 genes from WGCNA were compiled with the targets of miR-182-5p.

### qRT-PCR

Total RNA was extracted from NP tissues using Trizol (Thermo, IL, USA) as described previously^56^. CircRNA, miRNA and mRNA concentrations were determined using the ABI PRISM 7500 system (Applied Biosystems, CA, USA). GAPDH was used to normalize circRNA and mRNA expression levels, and U6 was used to normalize miRNA expression levels. All the primers used are listed in Supplementary Table S2.

### NPCs culture

NPCs were isolated from NP tissue of young Sprague-Dawley (SD) rats (100–150 g) as described previously^57^. NPCs were cultured in DMEM/F12 medium (Gibco, NY, USA) with 15% fetal bovine serum (Gibco, NY, USA). The second passage of cells was used in all experiments. To induce oxidative stress-induced mitochondrial dysfunction, cells were stimulated by 100 μM TBHP (Sigma, MO, USA) for 12 h. CCK-8 assay was used to monitor NPCs viability. The absorbance of the wells was measured using a microplate reader at 450 nm.

### Senescence-associated β-galactosidase staining

SA-β-Gal kit (Yeasen, Shanghai, China) was used for senescence-associated β-galactosidase staining. Three random microscopic fields per slide were observed under an BX53 microscope (Olympus, Tokyo, Japan).

### Cell transfection

*circ*ERCC2 vectors were constructed with amplified DNA fragments including the sequence of 13, 14, 15 exons of *ERCC2* gene with flanking introns containing complementary Alu elements (GeneChem, Shanghai, China). siRNAs for miR-182-5p (miR-182-5p-si) and SIRT1 (SIRT1-si) and scrambled siRNA were from GenePharma (Shanghai, China). NPCs (5 × 10^5^/well) were seeded in 6-well plates for 24 h and transfected with the vectors or siRNAs using Lipofectamine 3000 (Thermo, IL, USA) according to the manufacturer’s instructions. All sequences are listed in Supplementary Table S3.

### Dual-luciferase reporter assay

The 3′-UTR of SIRT1 gene or *circ*ERCC2 fragments were inserted into luciferase vector (Promega, WI, USA). NPCs were seeded in 96-well plates at 8 × 10^3^ cells per well, and co-transfected with the vectors, miR-182-5p and luciferase vector. The luciferase activity was measured using a luminometer (Promega, WI, USA) after 48 h.

### Western blot analysis

Total protein was extracted from NPCs using RIPA buffer with 1 mM phenylmethanesulfonylfluoride (Beyotime, Shanghai, China). The protein concentration was measured using a BCA protein assay kit (Thermo, IL, USA). Proteins were then separated by SDS-PAGE and transferred to polyvinylidene difluoride membranes (Bio-Rad, CA, USA). After blocking with 5% non-fat milk, the membranes were incubated overnight at 4 °C with primary antibodies (SIRT1, dilution: 1:1000; caspase3, dilution: 1:1000; caspase7, dilution: 1:1000; caspase9, dilution: 1:1000; MMP13, dilution: 1:1000; Collagen II, dilution: 1:1000; PINK1, dilution: 1:1000; Parkin, dilution: 1:500; p62, dilution: 1:500; LC3II/I, dilution: 1:1000; GAPDH, dilution: 1:1000, all from Abcam, Cambridge, UK), followed by incubation with secondary antibody. Finally, the intensities of the protein bands were quantified with Image Lab 3.0 software (Bio-Rad, CA, USA).

### RNA fluorescent in situ hybridization (FISH)

FISH was performed using the NP tissues and NPCs. Blue fluorescence (4,6-diamidino-2-phenylindole, DAPI) indicated cell nucleus; green fluorescence (Alexa 488) indicated *circ*ERCC2 and red fluorescence (Cy-5) indicated miR-182-5p. The images were then acquired under BX53 microscope (Olympus, Tokyo, Japan). The primer and prober sequences are listed in Supplementary Table S4.

### Flow cytometry

NPCs were seeded into 6-well plates at a density of 3 × 10^5^ cells per well. The rates of apoptosis were evaluated by flow cytometry using an AO2001-11A-H apoptosis detection kit (SUNGENE BIOTECH, Tianjin, China). The early apoptotic cells were Annexin V-APC+/7-AAD−, late apoptotic cells were Annexin V-APC+/7-AAD+, and normal cells were Annexin V-APC−/7-AAD−. The stained cells were analyzed using the FAC Scan flow cytometry (Beckman, CA, USA).

### TUNNEL staining

The apoptosis of NPCs was detected using TUNNEL staining kit (Yeasen, Shanghai, China). Three random microscopic fields per slide were examined under BX53 microscope (Olympus, Tokyo, Japan).

### Rat model of IVDD

Thirty-two adult female SD rats (200–250 g) were obtained from the Experimental Animal Institute of Shanghai University and housed in a controlled environment under standard conditions temperature and 12 h light and dark cycle. The rats were randomly divided into four groups, the control group (eight females), IVDD (no injection) group (eight females), *circ*ERCC2 group (eight females) and *circ*ERCC2-mut group (eight females). All rats were anaesthetized by intraperitoneal injection of 10% chloral hydrate (40 mg/kg). The model of IVDD was established as described previously^58^. Briefly, the coccygeal intervertebral spaces of Co7–8 were selected for the surgery. The tail discs of the rats were punctured with 18 G needles. The needles were retained in the discs for 1 min. Then *circ*ERCC2 and *circ*ERCC2-mut groups were intraperitoneally injected with *circ*ERCC2 or *circ*ERCC2-mut every week until they were sacrificed at 8 weeks.

### MRI examination

After 8 weeks of needle puncture, all rats were anaesthetized by intraperitoneal injection of 10% chloral hydrate (40 mg/kg). Sagittal T2-weighted images were chosen using a 7.0-T MR (Philips Intera Achieva 7.0 MR). The MRI images were evaluated by three orthopedic researchers. MRI grade was a 5-scale grading system according to the Pfirrmann grade^59^.

### Histological evaluation

The rats were sacrificed by intraperitoneal injection of over-dose of 10% chloral hydrate 8 weeks after needle puncture, and the punctured and non-punctured tails were collected. The tissues were fixed in 10% neutral-buffered formalin for 1 week, decalcified in EDTA for 3 weeks, and embedded in paraffin. The tissues were then cut into 5 μm sections. Then, the sections were stained with hematoxylin and eosin (H&E) and Safranin-O/fast green.

### Immunofluorescence staining

For immunofluorescence staining, the sections embedded in paraffin were deparaffinized and rehydrated. The sections were microwaved in 0.01 mol/L sodium citrate for 15 min, then incubated overnight with primary antibody at 4 °C (MMP13, dilution 1:200; Collagen II, dilution 1:200; all from Abcam, Cambridge, UK), followed by incubation with secondary antibody for 1 h. NPCs were washed with PBS for three times, Then, the cells were fixed with 4% paraformaldehyde for 15 min, permeabilized with 0.5% Triton X-100 for 20 min. The cells were then blocked with 1% goat serum albumin for 1 h, and incubated overnight with primary antibody at 4 °C (SIRT1, dilution 1:200; LC3B, dilution 1:200; TOMM20, dilution 1:200; all from Abcam, Cambridge, UK), followed by incubation with secondary antibody for 1 h. The nuclei were stained with DAPI for 5 min. Finally, the sections or cells were photographed under BX53 microscope (Olympus, Tokyo, Japan).

### Statistical analysis

All data are expressed as mean ± SD. The Shapiro-Wilk test was adopted to verify data distribution and the Levene test was used to test equality of variances. The data were analyzed by unpaired two-tailed student’s *t*-test (normal distribution and equal variances), Welch *t*’-test (unequal variances) or Mann-Whitney *U* test (non-normal distribution). Statistical analyses were performed using statistical software programs SPSS 24.0 (IBM, NY, USA) and GraphPad Prism 7 (GraphPad, CA, USA). *p* < 0.05 was considered significant.

## Supplementary information

Supplementary Figure 1

Supplementary Table S1

Supplementary Table S2

Supplementary Table S3

Supplementary Table S4

Supplementary information

Supplementary figure and table legends
