# Supplementary figures and images for: CircERCC2 ameliorated intervertebral disc degeneration by regulating mitophagy and apoptosis through miR-182-5p/SIRT1 axis
Source: Cell Death Dis. 2019 Oct 3;10(10):751. doi: 10.1038/s41419-019-1978-2 (PMC6776655; doi:10.1038/s41419-019-1978-2)

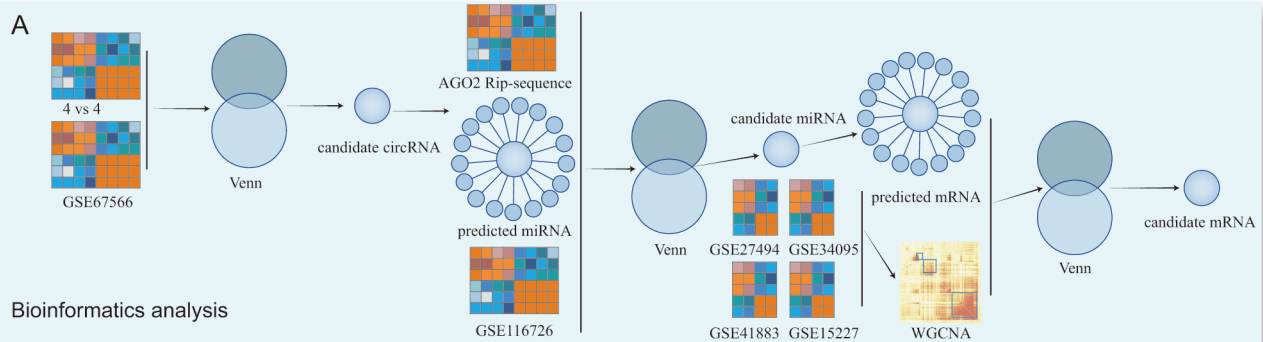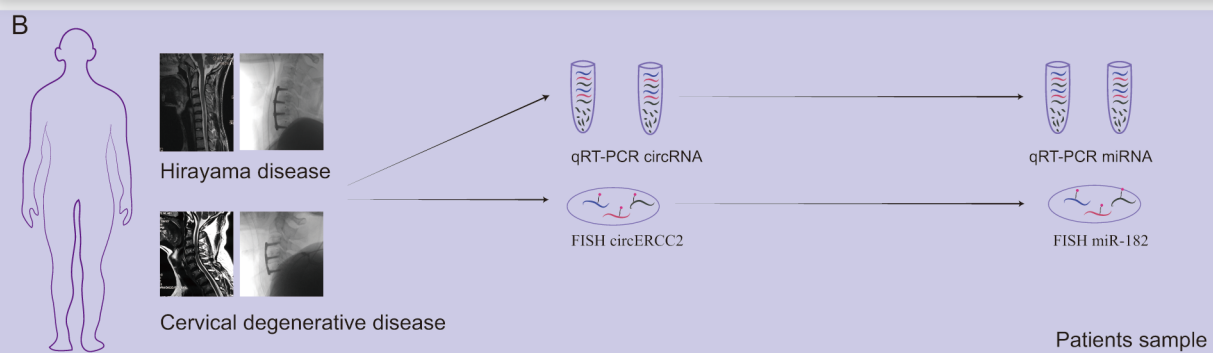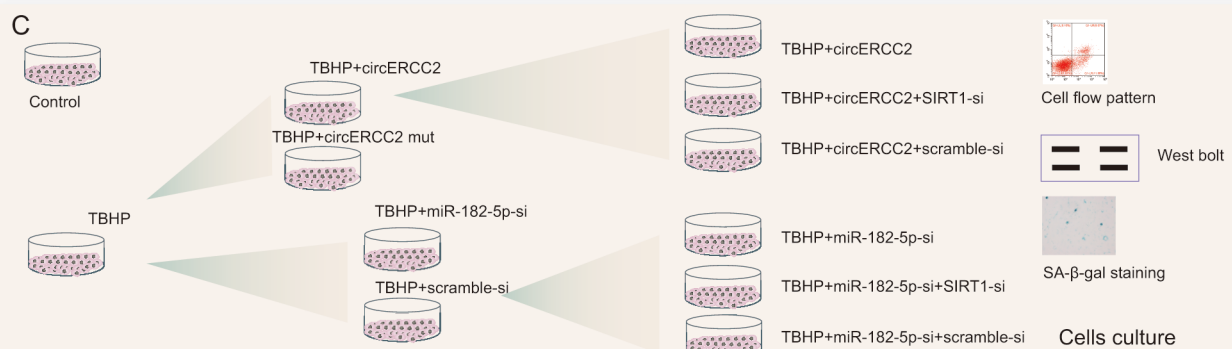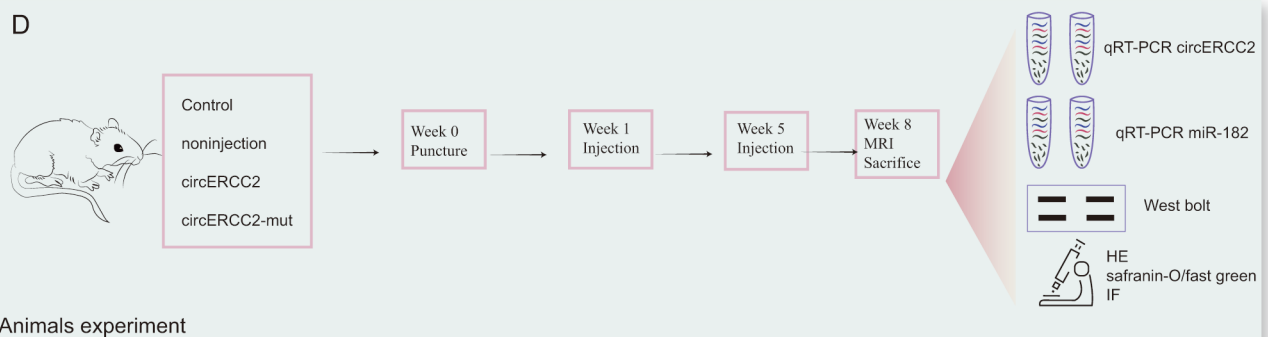

Supplement: Supplementary file 1 — Supplementary Figure 1 [file 41419_2019_1978_MOESM1_ESM.pdf]
