# Supplementary material for: CircERCC2 ameliorated intervertebral disc degeneration by regulating mitophagy and apoptosis through miR-182-5p/SIRT1 axis
Source: Cell Death Dis. 2019 Oct 3;10(10):751. doi: 10.1038/s41419-019-1978-2 (PMC6776655; doi:10.1038/s41419-019-1978-2)
Supplement: Supplementary file 2 — Supplementary Table S1 [file 41419_2019_1978_MOESM2_ESM.docx]

| Number | Sex | Age | Level | Pathology | Pfirrmann |
| --- | --- | --- | --- | --- | --- |
| 1 | F | 21 | C5/C6, C6/C7 | Hirayama disease | I |
| 2 | M | 16 | C4/C5, C5/C6, C6/C7 | Hirayama disease | II |
| 3 | M | 18 | C4/C5, C5/C6 | Hirayama disease | I |
| 4 | M | 17 | C4/C5, C5/C6, C6/C7 | Hirayama disease | I |
| 5 | F | 23 | C4/C5, C5/C6, C6/C7 | Hirayama disease | I |
| 6 | F | 21 | C4/C5, C5/C6, C6/C7 | Hirayama disease | I |
| 7 | F | 22 | C4/C5, C5/C6, C6/C7 | Hirayama disease | I |
| 8 | F | 17 | C4/C5, C5/C6 | Hirayama disease | II |
| 9 | M | 22 | C4/C5, C5/C6 | Hirayama disease | I |
| 10 | M | 31 | C4/C5, C5/C6 | Hirayama disease | II |
| 11 | F | 22 | C4/C5, C5/C6 | Hirayama disease | II |
| 12 | M | 18 | C4/C5, C5/C6, C6/C7 | Hirayama disease | I |
| 13 | M | 17 | C4/C5, C5/C6, C6/C7 | Hirayama disease | I |
| 14 | M | 18 | C4/C5, C5/C6, C6/C7 | Hirayama disease | I |
| 15 | M | 19 | C4/C5, C5/C6, C6/C7 | Hirayama disease | I |
| 16 | M | 21 | C4/C5, C5/C6, C6/C7 | Hirayama disease | II |
| 17 | F | 62 | C4/C5, C5/C6 | Cervical Myelopathy | IV |
| 18 | M | 61 | C4/C5, C5/C6 | Cervical Myelopathy | IV |
| 19 | F | 66 | C4/C5, C5/C6 | Cervical Myelopathy | IV |
| 20 | M | 76 | C4/C5 | Cervical Myelopathy | IV |
| 21 | F | 78 | C5/C6 | Cervical Myelopathy | V |
| 22 | M | 65 | C4/C5, C5/C6 | Cervical Myelopathy | V |
| 23 | M | 77 | C5/C6 | Cervical Myelopathy | V |
| 24 | F | 74 | C5/C6 | Cervical Myelopathy | IV |
| 25 | M | 78 | C5/C6 | Cervical Myelopathy | IV |
| 26 | M | 75 | C5/C6 | Cervical Myelopathy | IV |
| 27 | F | 72 | C5/C6 | Cervical Myelopathy | V |
| 28 | M | 69 | C5/C6 | Cervical Myelopathy | V |
| 29 | F | 72 | C5/C6 | Cervical Myelopathy | V |
| 30 | F | 76 | C4/C5, C5/C6 | Cervical Myelopathy | V |
| 31 | F | 77 | C4/C5, C5/C6 | Cervical Myelopathy | IV |
| 32 | M | 72 | C5/C6 | Cervical Myelopathy | IV |

F: Female

M: Male
