# Supplementary material for: CircERCC2 ameliorated intervertebral disc degeneration by regulating mitophagy and apoptosis through miR-182-5p/SIRT1 axis
Source: Cell Death Dis. 2019 Oct 3;10(10):751. doi: 10.1038/s41419-019-1978-2 (PMC6776655; doi:10.1038/s41419-019-1978-2)
Supplement: Supplementary file 3 — Supplementary Table S2 [file 41419_2019_1978_MOESM3_ESM.docx]

| hsa-circ-0007618 | F: GCCACCAGGACCCTAACTCAA  R: TTCTCTGCAATGACCTGGGAC |
| --- | --- |
| hsa-circ-0015787 | F: CTGCTGGACTACTGCTGCATT  R: GCAGCATACTGGCATACAGCT |
| hsa-circ-0000894 | F: CTCCCATCTGGTCCAGCATTC  R: TCCAAAGGCTCCCTCACAAGT |
| hsa-circ-0012107 | F: GCCATCTTCCCCCGCTCAC  R: GTCCTCGCTCAGTGACTTCAAAA |
| hsa-circ-0004711 | F: AGCCTGCCTTTCCAGATCCAT  R: CTGGTGTGCTGTGCTTCCAAT |
| hsa-circ-0003502 | F: ATCTTGATAGTCATTTTCATGGTGA  R: AGGGAGGTCATCAAATGTCTGAGAT |
| hsa-circ-0092314 | F: TCCAGTCCAGATCGTCCATGC  R: AACCAAAGTGCCAGCCACAAG |
| hsa-circ-0051470 | F: TGCCTCTGCCCTATGGCTC  R: CAGGATGGGGTTGGCAATGG |
| hsa-circ-0005795 | F: GAGGTGTTTGGTGCAGAAGTGA  R: CAAGCATTGAGTCCAGGGAGG |
| has-miR-182-5p | F: ATCACTTTTGGCAATGGTAGAACT  R: TATGGTTTTGACGACTGTGTGAT |
| GAPDH | F: ATCACTGCCACCCAGAAG  R: TCCACGACGGACACACATTG |
| U6 | F: ATTGGAACGATACAGAGAAGATT  R: GGAACGCTTCACGAATTTG |
