# Supplementary material for: CircERCC2 ameliorated intervertebral disc degeneration by regulating mitophagy and apoptosis through miR-182-5p/SIRT1 axis
Source: Cell Death Dis. 2019 Oct 3;10(10):751. doi: 10.1038/s41419-019-1978-2 (PMC6776655; doi:10.1038/s41419-019-1978-2)
Supplement: Supplementary file 4 — Supplementary Table S3 [file 41419_2019_1978_MOESM4_ESM.docx]

***circ*ERCC2 vector**

F ACCCCAAGATCCTGGACTTC

R TCAAATACGGGTTTGATGGC

**miR-182-5p inhibitor**

5’-CGGUGUGAGUUCUACCAUUGCCAAA-3’

**miR inhibitor N.C.**

5’-CAGUACUUUUGUGUAGUACAA-3’

**SIRT1-si**

5’-CCCUGUAAAGCUUUCAGAATTUUCUGAAAGCUUUACAGGGTT-3’

**Negative control**

Sense 5’-UUCUCCGAACGUGUCACGUTT-3’

Antisense 5’-ACGUGACACGUUCGGAGAATT-3’

**CircVMA21 sequence**

CGCCCCGGTTTTCCCCTGGAAATTCCATATTGGCACGCATTCTATTGGCTGAGCTGCGTTCTACGTGGGTATAAGAGGCGCGACCAGCGTCGGTACCGTCGCAGTCTTCGGTCTGACCACCGTAGAACGCAGATCGAATTAAGCTTGGGCTGCAGGTCGACTCTAGAGGATCCCCGGGTACCGGTTAATACGACTCACTATAGGGAGACCCAAGCTGGCTAGCTCCTACATGCGCTCAAGAAAACAATGCCTGCTGTGATTTCTAGAATAAATGAATGTGAACCACAGTTCCTTTACTTGACTAACAGAGAAAGTTTAAATATCAACCTAGTCATTAACCACAGTTATTAAACCACGTTAAACAACCAGCAAGGGGTTAAGAAAGAAAGTTGCTATGTTTTTTCTTTCATTGCTGAATGAGTCTAACTTAGTTACTGTATCAACCTTAATACAGAACATTGTTTGCATCTCAATGGTTCTCTAAAATTATTCGTTCATGGCTTGAGTTCTAAAATTAAACTATGTGGAGTCATGTCCAACCGCACAATGCATCTTTATGTGAAACTTGCTAGAGTTTTTGTTTTCCTTCTATGTAAAAGTCCAGTTGGGAAGCTTTATTTCTGATAGATTAAATGGTATAGGTCTTTCAGTTTTCTCTTCATTTCTGACAACTGAACTGCTCTCGCCTTGAACCTGTTTTGGCGGTACCAGGCTTCACCATCATCATCGAGCCCTTTGACGACAGAACCCCGACCATTGCCAACCCCATCCTGCACTTCAGCTGCATGGACGCCTCGCTGGCCATCAAACCCGTATTTGAGCGTTTCCAGTCTGTCATCATCACATCTGGGACACTGTCCCCGCTGGACATCTACCCCAAGATCCTGGACTTCCACCCCGTCACCATGGCAACCTTCACCATGACGCTGGCACGGGTCTGCCTCTGCCCTATGGTGGATCCGCCAAAACAGGTTCAAGGCGAGAGCAGTTCAGTTGTCAGAAATGAAGAGAAAACTGAAAGACCTATACCATTTAATCTATCAGAAATAAAGCTTCCCAACTGGACTTTTACATAGAAGGAAAACAAAAACTCTAGCAAGTTTCACATAAAGATGCATTGTGCGGTTGGACATGACTCCACATAGTTTAATTTTAGAACTCAAGCCATGAACGAATAATTTTAGAGAACCATTGAGATGCAAACAATGTTCTGTATTAAGGTTGATACAGTAACTAAGTTAGACTCATTCAGCAATGAAAGAAAAAACATAGCAACTTTCTTTCTTAACCCCTTGCTGGTTGTTTAACGTGGTTTAATAACTGGACTTCCTCTCGAGTCTAGAGGGCCCGTTTAAACCCGCTGATCAGCCTCGCTAGCTAACTGTGGAATGTGTGTCAGTTAGGGTGTGGAAAGTCCCCAGGCTCCCCAGCAGGCAGAAGTATGCAAAGCATGCATCTCAATTAGTCAGCAACCAGGTGTGGAAAGTCCCCAGGCTCCCCAGCAGGCAGAAGTATGCAAAGCATGCATCTCAATTAGTCAGCAACCATAGTCCCGCCCCTAACTCCGCCCATCCCGCCCCTAACTCCGCCCAGTTCCGCCCATTCTCCGCCCCATGGCTGACTAATTTTTTTTAT
