# Supplementary material for: CircERCC2 ameliorated intervertebral disc degeneration by regulating mitophagy and apoptosis through miR-182-5p/SIRT1 axis
Source: Cell Death Dis. 2019 Oct 3;10(10):751. doi: 10.1038/s41419-019-1978-2 (PMC6776655; doi:10.1038/s41419-019-1978-2)
Supplement: Supplementary file 5 — Supplementary Table S4 [file 41419_2019_1978_MOESM5_ESM.docx]

hsa_circ_0051470 probe1 5'ATGATGGTGAAGCCATAGGGCAGAG3' 5'FAM

hsa_circ_0051470 probe2 5'GATGATGATGGTGAAGCCATAGGGC3' 5'FAM

hsa_circ_0051470 probe3 5'TGGTGAAGCCATAGGGCAGAGGCAG3' 5'FAM

miR-182-5p 5' CGGTGTGAGTTCTACCATTGCCAAA 3' 5'CY3
