# Supplementary material for: CircERCC2 ameliorated intervertebral disc degeneration by regulating mitophagy and apoptosis through miR-182-5p/SIRT1 axis
Source: Cell Death Dis. 2019 Oct 3;10(10):751. doi: 10.1038/s41419-019-1978-2 (PMC6776655; doi:10.1038/s41419-019-1978-2)
Supplement: Supplementary file 7 — Supplementary figure and table legends [file 41419_2019_1978_MOESM7_ESM.docx]

**Supplementary figure legends**

**Supplemental Figure 1.** Schematic diagrams of the experimental design and protocol. (A) Bioinformatics analysis. (B) Patients sample collection. (C) Cells culture experiment. (D) Rat model experiment.

**Table S1.** Demographic data on surgical disc patients.

**Table S2.** Primers used for qRT-PCR analysis.

**Table S3. S**equences of circRNA vector and siRNAs.

**Table S4.** The probe sequences of *circ*ERCC2 and miR-182-5p in FISH.
